# Transition to adult healthcare for immigrant youth: Practice recommendations

**DOI:** 10.1016/j.hctj.2024.100079

**Published:** 2024-11-05

**Authors:** Andrew S. Mackie, Mia Tulli-Shah, Alyssa Chappell, Michael Kariwo, Siciida Ibrahim, Bukola Salami

**Affiliations:** a Department of Pediatrics, University of Alberta, 11405-87 Avenue, Edmonton, Alberta T6G 1C9, Canada; b Faculty of Nursing, University of Alberta, Faculty of Nursing, University of Alberta, 11405-87 Avenue, Edmonton, Alberta T6G 1C9, Canada; c Faculty of Education, University of Alberta, 11210 - 87 Avenue, Edmonton, Alberta T6G 2G5, Canada

**Keywords:** Healthcare transition, Adolescence, Migration, Newcomers, Immigrants, Policy and practice

## Abstract

**Introduction:**

The transition from pediatric to adult healthcare is challenging for adolescents and young adults (AYA) with pediatric-onset chronic health conditions. Although barriers faced by AYA during transition are well-documented, previous studies have not considered how migration and settlement impact patient and family experiences.

**Objectives:**

To fill this gap, we conducted a qualitative descriptive study to explore the recommendations for policy and practice from the perspectives of immigrant and refugee AYA living with chronic health conditions in Canada as they transition from pediatric to adult healthcare. We also sought the perspectives of their parents/caregivers and service providers.

**Methods:**

Semi-structured individual interviews and focus groups were conducted with 20 AYA, 14 caregivers, and 5 service providers. AYA were 1st or 2nd generation immigrants to Canada, aged 16–25, with childhood-onset chronic health conditions. Parents or caregivers were 1st generation immigrants, having children with chronic health conditions. Service providers delivered healthcare or other services to immigrant populations.

**Results:**

Recommendations to improve the transition process and mitigate barriers to care included providing more accessible information about patients’ health conditions prior to transition, moving the age of transfer beyond age 18, establishing a centralized online health portal for patients who are transitioning to the adult system, providing family case workers, increasing language support, and increasing cross-sector support.

**Conclusion:**

A broad range of recommendations aimed at improving the transition process were provided. Future interventions to support transition from pediatric to adult care for immigrants and refugees should incorporate these recommendations.

## Highlights

• Immigrant youth living chronic health conditions face major transition hurdles
• Barriers to transition include language barriers, discrimination, and finances
• Recommendations include providing increased information about patients’ health conditions prior to transition, moving the age of transition later, a centralized online health portal for patients who are transitioning to the adult system, the provision of family case workers, increasing language support, and increasing cross-sector support

## Graphical abstract

**Figure:**
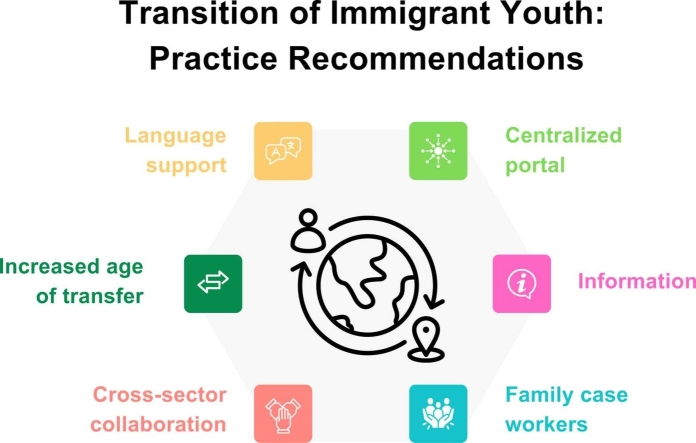

## 1 Introduction

Ten to fifteen percent of adolescents in North America live with one or more chronic health conditions and require transition from pediatric to adult-oriented services. However, many pediatric programs lack the resources required to facilitate transition.^1^, ^2^ These resources include multidisciplinary teams in the clinical setting with staff (e.g., nurses, social workers, psychologists, physicians) that can teach adolescents and young adults (AYA) about their health condition and coach self-management skills, address social determinants of health and/or mental health co-morbid conditions that result in barriers to receiving care, and evaluate transition readiness throughout adolescence. In addition to human resources, space in clinical settings is needed to work 1-on-1 with AYA.

Blum et al. defined transition (p. 570, 1993) as “the purposeful, planned movement of adolescents and young adults with chronic physical and medical conditions from child-centered to adult-oriented healthcare systems.^3^” Within this definition successful transition involves the development of self-management skills and independence.^4^ Family member roles also ideally shift as AYA develop greater autonomy in their medical care. In contrast, the term “transfer” refers to the point in time when the patient’s care and their medical records are moved from the pediatric to the adult providers. Transfer is part of transition, but the two are not synonymous.

Transition is a challenging and stressful process for AYA and their families,^5^, ^6^ and a period of medical risk. Transition-age youth with chronic health conditions experience an increase in emergency room visits^7^ and hospitalizations, gaps in receiving ambulatory care,^8^ decreased health status,^9^ poor mental health,^10^ noncompliance with treatment plans,^11^ as well as increased caregiver burden.^12^ The consequences of transition may be heightened among immigrants, who face additional barriers. For example, newcomers to Canada may have limited language and literacy skills, lack of familiarity with the healthcare system, fear and distrust of a new healthcare system, complex health insurance eligibility, low socioeconomic status, poor housing, and limited pre-arrival care.^13^, ^14^ Factors related to gender and culture may also complicate the transition process. Both first- and second-generation immigrant populations experience complexities related to their health status such as income, housing, socioeconomic status, and access to healthcare.^15^

There were approximately 1328,240 recent immigrants or refugees who entered Canada between 2016 and 2021, of whom 20 % (271,950) were children under age 15.^16^ Children with an immigrant background (first- or second-generation) comprise nearly 38 % of children in the Canadian population.^17^ Depending on their country of origin, chronic health conditions including sickle cell disease, human immunodeficiency virus, chronic hepatitis B, and post-traumatic stress disorder are more common in immigrant than non-immigrant youth.^14^

Our team previously published data regarding the barriers and facilitators of transition of immigrant and refugee youth.^18^ Barriers to successful transition include a lack of family experience in Canada, language barriers, discrimination, financial strain, health stigma, and long wait times for service access.^18^ Facilitators of successful transition include the ability of AYA to be independent, AYA ability to act as cultural bridges within their families, and cross-sector support.^18^ The objective of the current manuscript is to describe recommendations to improve policy and practice surrounding transition for this population, from the perspective of AYA, their parents/caregivers, and service providers.

## 2 Material and Methods

### 2.1 Design

This is a secondary analysis of interviews previously conducted by our team. We undertook a qualitative descriptive study in which we conducted semi-structured interviews with first- and second-generation immigrant AYA living with chronic health conditions, and parents or caregivers. A first-generation immigrant was defined as someone born outside of Canada, including refugees, and second-generation as someone with at least one parent born outside of Canada. Five service providers (three physicians, one social worker, and one community service worker) who provide services to immigrant AYA with chronic health conditions were also interviewed. All AYA and caregivers were invited to participate in semi-structured interviews and a focus group. Focus groups were optional, as we recognized that not all participants would be comfortable participating in a group event. Service providers were enrolled for semi-structured interviews only.

### 2.2 Participants and recruitment

Inclusion criteria for participation among AYA participants were a) being a first or second-generation immigrant in Canada, b) aged between 16 and 25 years, and c) living with a pediatric-onset chronic health condition. For parents and caregivers, inclusion criteria were a) being a first-generation immigrant to Canada, and b) having a child aged 16–25 living with a pediatric onset chronic health condition. Service providers were included if they worked in healthcare settings or for organizations that provide community services to immigrant AYA and their families, though they may not have provided care to the AYA participants in this study.

Transfer to adult care in Canada typically occurs at age 18. Successful transition requires knowledge of the condition, self-management and self-advocacy skills, and the ability to navigate the healthcare system^19^ which may take several years to achieve. Therefore, we targeted youth aged 16–25 to include participants across the range of the transition continuum.

Recruitment took place across Canada. Strategies included: advertising the study with flyers posted in clinical settings that serve a large number of immigrant or refugee AYA and families; approaching partner organizations in the community service sector that work with immigrant populations to assist with recruitment of participants; secondary recruitment of participants in the control arm of the Transition Navigator Trial^20^ (a large transition related research study conducted across the province of Alberta, Canada); advertising via social media (Instagram, Facebook and Twitter); and word of mouth. Purposive sampling was used to ensure that participants were selected based on maximum variation (i.e., medical conditions and demographics including ethnicity, race, and gender). Recruitment of participants concluded when data saturation was reached.

### 2.3 Data collection and analysis

Data collection occurred from 2020 to 2022. Interviews were conducted over Zoom™ (except for one interview that took place over the phone) and took approximately one hour. Focus groups had 3–7 participants each and were moderated by a trained qualitative researcher and an assistant. Focus groups took approximately two hours. Audio recordings of interviews and focus groups were transcribed verbatim prior to analysis. All interview and focus group quotes have been attributed a participant pseudonym to maintain confidentiality. Migration path was asked of the caregiver participants but not of AYA participants.

For AYA participants who were unable to participate independently due to developmental delay, we interviewed a parent or caregiver only. We did not exclude participants based on language of preference. During the informed consent process our team assessed conversational level of English to ensure participants understood the study and could participate in a subsequent interview and/or focus group. We did not complete a formal language assessment with participants. For participants who preferred interviews be conducted in a language other than English, interpreters were used. Only the English interpretation of these interviews was transcribed and analyzed.

We used NVivo 12 qualitative software and drew on Braun and Clark’s^21^ (2006) reiterative process for thematic analysis. This process demanded researchers first familiarize themselves with transcripts, and then create a coding framework which they revised and refined as data was coded. This allowed themes and patterns to develop from these codes.

### 2.4 Ethics approval

The study received approval from the University of Alberta research ethics board on October 21, 2019 and all participants provided informed consent prior to participation. This study was performed in compliance with relevant laws and institutional guidelines.

## 3 Results

A total of 20 AYA, 14 caregivers and 5 service providers participated in individual interviews. Twelve of the AYA and 4 parents also participated in a focus group. Most AYA participants (n=13) identified as girls or women, as did the parents and caregivers (n=11). All caregivers and 11 youth were first generation immigrants. The remaining 9 youth were second generation immigrants.

AYA participants had a mean number of years in Canada of 12.4, and SD = 7.9. Caregivers had a mean number of years in Canada of 15.2 and SD = 13.0. Participants’ or their parents’ countries of origin were Australia, Bangladesh, Brazil, China, Colombia, Egypt, England, Ethiopia, Guyana, India, Indonesia, Iran, Jamaica, Kuwait, Lebanon, Mexico, Myanmar, Nigeria, Pakistan, Philippines, Syria, United States of America, Taiwan, and Zambia. In three families both AYA and their caregiver participated in separate interviews. In other families, either AYA or parent (but not both) participated. One participant required translation during both the informed consent process as well as during the individual interview. Native languages identified by youth and caregiver participants included Arabic, English, Indonesian, Mandarin, Spanish, and Tigrinya. With such heterogeneity of native languages and countries of origin, we did not compare findings based on these demographics. Demographic information can be found in Table 1.

**Table 1:** Demographic Information.

**Variable**	**AYA n = 20**	**Caregiver n=14**
**Age, years (mean ± SD)**	20.1 ± 2.6	
**Gender**		
Men	7 (35)	3 (21)
Women	13 (65)	11 (79)
Other	0 (0)	0 (0)
**Immigrant generation**		
First Generation	11 (55)	14 (100)
Second Generation	9 (45)	0 (0)
**Current immigration status**		
Canadian Citizen	16 (80)	8 (57)
Permanent Resident	3 (15)	5 (36)
International Student	1 (5)	0 (0)
Undisclosed	0 (0)	1 (7)
**Immigration path**		
Economic/skilled worker		5 (36)
Refugee		3 (21)
Temporary foreign worker		1 (7)
Other		3 (21)
Undisclosed		2 (14)
**Marital status**		
Married	0 (0)	11 (79)
Single	20 (100)	2 (14)
Undisclosed	0 (0)	1 (7)
**Education completed**		
High school or less	15 (75)	4 (28)
College certificate/diploma	1 (5)	4 (28)
University Degree	3 (15)	2 (14)
Graduate Degree	1 (5)	3 (21)
Undisclosed	0 (0)	1 (7)
**Youth has family doctor**	19 (95)	12 (86)
**Youth receives specialty care**	16 (80)	11 (79)
**Youth has transitioned to adult care**	14 (70)	5 (36)
**Youth has 2+ chronic health conditions**	6 (30)	4 (29)

*all values expressed as N (percentage) unless otherwise stated

AYA= adolescents and young adults

Many of the AYA had more than one chronic health condition. Chronic health conditions of the AYA participants varied widely, with the most prevalent being hematological and cardiovascular. Specific health conditions included: imperforate anus, megacolon, kidney disease, brain tumor, type 1 diabetes mellitus, bulimia, post-traumatic stress disorder, depression, anxiety, borderline personality disorder, Crohn’s disease, sickle cell anemia, gastrointestinal reflux disease, Tourette’s syndrome, global developmental delay, traumatic brain injury, cognitive impairment, autism spectrum disorder, polychondritis, cerebral palsy, chronic pain, epilepsy, scoliosis, asthma, congenital heart disease, postural hypotension, anemia, thrombocytopenia, VACTERL syndrome, beta thalassemia major, and obsessive compulsive disorder. At the time of their interview, 68 % of the AYA participants had entered the adult healthcare system; the remaining were still managed by pediatric healthcare providers. For the latter group we focused primarily on their sense of preparedness and anticipation of the upcoming transition.

Qualitative analysis of the data set illuminated three themes: barriers and facilitators of successful transition (previously published by our team^18^), and recommendations to policy makers and service providers to address challenges. Recommendations included providing accessible information prior to transition, moving the age of transfer later, developing a centralized online health portal for patients who are transitioning to the adult system, the provision of family case workers, increasing language support, and increasing cross-sector support.

### 3.1 Increased information about their condition and care moving forward prior to transition

Eight youth reported a lack of information about their transition prior to the move from pediatric to adult healthcare. They reported this made the change feel abrupt and uncertain. To mitigate against this abruptness, many recommended having accessible information about both their condition and their care moving forward in the adult system prior to their transfer of care. However, they also discussed the caveat that feeling overloaded with information can prevent them from understanding what is going on. Thus, there was a consensus for information to be introduced gradually over several years prior to transition. Additionally, most participants advocated that youth who are about to transition be introduced to their new doctors and that this introduction be facilitated by their current pediatric providers.

I think kind of like a bridging between both sides, where the adult healthcare providers can meet at least the patient once, just so the patient knows who they are and who they’re reaching out to, would just help as an introduction into adult healthcare [Olivia, Youth].

Olivia’s use of the word “bridging” shows the importance of connecting pediatric and adult systems for youth about to move from one to the other. She advised patients who are entering the adult system should do so while they still have access to familiar pediatric providers.

### 3.2 Moving age of transfer later

The idea of moving the age of transfer later was mixed between youth and caregivers. Many of each group voiced feeling that age 18 is a good time to move on to the adult healthcare system, explaining it was time for patients to fully enter the adult world. Others advised 18 as too early for such a change, explaining a move at 21 or 25 would be more appropriate. In one case a father asserted that a 20-year-old who grew up with a chronic condition and who had spent time in the pediatric system is less prepared to navigate the adult system than someone of the same age who experienced a chronic health condition onset after adulthood, and thus, those who grew up in the pediatric system should be afforded more time before entering adult care. Some lamented the fact that youth brains are still developing into the early twenties. Others, especially youth, reported that age 18 is already a period of such large change, usually including the exit from secondary school and often the start of post-secondary. They felt that adding transfer of care at such a turbulent time creates too much stress and it would be better to stagger big life changes.

I think 21 is a good – you know, at that age you’ve lived a little bit. You’ve experienced some things, and you’re at a maturity level that you can probably deal with it better, I would think [Lincoln, Caregiver].

Though not all youth or caregivers agreed with the need to delay the age of transfer, those who did, advised sometime between the ages of 20–25 may enable a better experience.

One healthcare provider participant explained their job was to meet with youth experiencing complex care needs prior to their transfer to gauge transition readiness across multiple measurements. They explained this approach offered opportunities to more successfully support the youth through their transition because they may be ready to transition in some aspects but need assistance in others.

[The meeting] takes about an hour, where we go through kind of their lives and what’s going on for them, their medical condition, and assess different areas of transition readiness, so self-management, instrumental support, systems navigation, all those kinds of things, identify what needs to be done, any gaps or areas for growth, and then work with the youth to develop plans around accomplishing those things, or making behaviour changes to accomplish their goals, and then providing support over the transition period, so like a couple of years, while they get settled into adult healthcare [Jayne, Healthcare provider].

Such an approach may allow clinicians to know when a young adult is ready to have their care transferred, providing a personalized approach to care.

### 3.3 Centralized portal

As touched on above, many youth participants explained how a lack of helpful information often coupled with feeling inundated with material. Several reported they were not able to remember useful parts of this information overload and did not know where to find information or resources in the moment when they needed it afterwards. In the first youth focus group, participants discussed possibilities for one centralized patient portal where they could view information about upcoming appointments, locations, next steps, etc., at their leisure.

Like when I go to my family doctor, they have like a portal where I can see my appointments, or my medication. But I don’t think there is anything like that for people who are specialists, like higher up doctors and stuff like that [James, Youth].

Though two participants who had moved from pediatric care noted they had had access to such a portal, most participants did not for information related to their transition. James and others reported frustration that they had no access to such a centralized portal to help them navigate their transition.

### 3.4 Increased language support

Language barriers were cited as a key barrier preventing access to healthcare and successful transition. Participants explained that language barriers are more complicated than simple English skills and can have implications beyond understanding medical information. In many families children were at some point responsible for interpreting for their parents. In others, one parent had to translate for the other, creating imbalance between them and sometimes leading to family conflict.

Oh, if we can have somebody to translate at the moment, that will be wonderful. Not your husband, not your family, just somebody who can understand very good the terms, medical situation, like somebody who can translate very well, doctors translate, everything [Paula, Caregiver].

As Paula suggests, many participants reported a need for interpretation during appointments. Others, including Dolores, also note the need for support beyond appointments.

It was also suggested that providing written patient facing materials in multiple languages (other than the official languages of English or French) may be helpful to AYA and their families in understanding their chronic health condition in their own cultural context:

Yeah, …. I think one thing that I’m really hoping we would get better at is having like written summaries of things in different languages, like even for families to understand what is the diagnosis or something, just to, you know, what is ADHD in, you know, a different language? How do you say “autism” in your own language? Like and there may not be that language around it, depending on the cultural backgrounds of families, but just to – just for people to understand it within their own cultural context I think is really important, and I think that’s something that we’re not as good at. You can find resources in Spanish and in French, maybe, but apart from that, it can be quite challenging to find different language resources. – [Christina, Healthcare provider]

### 3.5 Provide family case workers

An emphasis on cross-sector support and the recognition of families' unique needs led several participants to recommend case workers support newcomer families of youth with chronic health conditions. They advised case workers to support families long-term so they can build relationships that offer them insight into their short and long-term needs. This may mitigate against many barriers to healthcare access and transition including lack of family experience in Canada, lack of cultural competency on the part of providers, and language barriers, which are often more nuanced than basic language skills and may be shaped by stress, situation-specific emotion, and cultural approaches to authority. As Delores suggests:

A lot of cultures will not ask questions. They will go to the appointment, they will let the doctors talk, or the medical practitioners talk, and then they leave…And if they are being invited to say, “Do you *have* any questions?” the answer is, “No.” The doctor’s going to say, “Do you *understand* what I just said? Or do you understand what is going to happen?” The answer is always, “Yes” [Delores, Caregiver].

She went on to explain how such a deference to authority can give the impression patients and their families have higher language skills and understanding of their care than they actually do.

Oh, you can speak English. Well, they can have an understanding. They don’t *need* a case worker.” And that’s where the big [issues] come into play. Even English speakers, when they are faced with trauma, they’re not – their frontal lobe is not going to work when they’re trying to just exist in the time of that trauma, right? So, for me, I researched a lot. So, I had that background, and even with that background, I still fell into that panic mode, into that, “I did not understand. I’m sorry, what did you say yesterday, what was going to happen?” [Delores, Caregiver].

Case workers who know the family may help them ask questions and can clarify information afterwards if parents are unable to remember details. Case workers, social workers or patient navigators may also help AYA and their families to navigate the healthcare system, as Christina suggests:

I think like having a social worker is key. Like I feel like the medical piece is one thing, but having a – having social workers, dietitians, like these allied health providers who can kind of help bridge that gap, like navigators, people who understand the health system, who may not be necessarily healthcare workers but maybe are community-based volunteers or employees within things like immigrant associations and stuff like that, who can help families navigate. Because it’s really about the journey. Like it – you know, all the pieces are there, but then how do you really move from one place to another? So it’s almost like less the healthcare team; it’s more these people in between who can help families with the navigation. – [Christina, Healthcare provider]

### 3.6 Increased cross-sector collaboration

Increased cross-sector collaboration is connected to the theme of family case workers, as case workers may not be limited to one sector. Instead, their support can cross healthcare, settlement, education, and housing challenges. As noted above, nine youth and three caregivers discussed the importance of cross-sector support. In cases where patients received support from a combination of healthcare providers, schools, social workers, and settlement workers, they reported this was an important facilitator in healthcare access and successful transition. However, many explained cross-sector support is limited and they do not see significant collaboration between service sectors.

I feel it could get better if the clinicians would collaborate a little bit with the university and the education system after you graduate high school. Because like we’re still adults. Like we still want to have jobs and successful careers, just like everyone else. Like why put us – why just leave us hanging there? [Sofia, Youth].

Participants reported a need for increased service provision and collaboration of those services across service sectors. Several recommended increased educational support that is extended into postsecondary education.

One service provider interview echoed the need for greater cross-sector collaboration when supporting newcomer youth.

We usually tend to take really good care of the babies. We think about the adults, because we think that the adults are very essential to be able to carry on with the family, but we forgot about the youth, and the youth is kind of in the middle, you know, being forgotten many times… we really need to pay attention to these population and do more, and I believe that if we strengthen this population, we are going to have good results in the future [Arooj, Care support worker].

Arooj spoke about the focus on babies and adults throughout both the healthcare and immigration sectors. She discussed her own experiences as a child newcomer in Canada, as well as her clients’ needs, explaining the need for holistic support and focus on youth who are often tasked with significant family responsibilities in immigrant and refugee families. Thus, supporting youth across service sectors may aid in transition and benefit family units.

## 4 Discussion

Participant recommendations to make transition more successful included having accessible information about their condition and future care, moving the age of transfer later, having a centralized online health portal for patients who are transitioning to the adult system, the provision of family case workers, increasing language support, and increasing cross-sector support. Some of these recommendations, especially providing accessible information prior to transition, moving the age of transfer later, and providing a centralized online health portal to patients, would likely benefit both immigrant and non-immigrant youth. Others, such as increasing language support, providing family case workers, and increasing cross-sector support would likely offer uniquely important benefit to immigrant and refugee patients and their families who may be contending with health challenges shaped by migration and settlement experiences, as well as who may be navigating challenges across multiple systems and policy sectors all at once.

Our findings add greater depth to prior studies about transition among adolescents that have not been specific to immigrant youth. For example, Mahan et al.^22^ (2017) advise transition to be organized through three stages. First, providers should set the stage by initiating transition support and assessing patient readiness.^22^ Youth participants in this study explained a need for more information prior to their transitions while also reporting being overloaded with information can cause them to freeze and lose focus. Many advised for more gradual information provided over several years prior to exit from the pediatric system. By gradually setting the stage for the move to adult healthcare, providers may be best able to strike a balance in offering enough information without overloading youth, causing them to feel overwhelmed. For all AYA, having an increased understanding of their health condition and health literacy prior to transition to adult care would be beneficial.^9^

During this first stage, providers may assess readiness of patients on a case-by-case basis. The idea of moving transfer to an older age was discussed with mixed perspectives. Slightly more than half of our participants recommended such a shift to at least the age 21, with some advising the move could be made up to 25. Others reported 18 a good age to shift systems as they felt it more appropriate for adults to leave a system meant for children. While all participants preparing for an upcoming transfer reported some apprehension, some of them did feel ready to move systems. Prior work has shown that readiness may be dependent on how formally planned the transition has been.^23^ However, psychological science also supports an increase in age of transfer. Though cognitive capacity (i.e., the ability to make deliberate decisions) reach adult levels around the age of 16, psychosocial maturity often does not until the early 20 s^24^ This means that though 18-year olds may be able to understand and make decisions they may be unable to do so during stressful and emotional situations until much later. Although it has been thought that age 18 is an appropriate age for transfer, there has been some evidence to support a later age of transfer in improving health outcomes for all AYA.^25^

As a second stage, Mahan et al.^22^ (2017) advised providers enable patients by offering ongoing transition support. As one tool through which to offer this support, participants touted possibilities for technology in aiding their transition. For example, many participants lamented the lack of a centralized online portal to review appointments, location of doctors, and medical information after they enter the adult system. Though there is limited knowledge about the effectiveness of mobile apps in facilitating transition of healthcare, data does suggest such tools can aid in increasing knowledge of the health condition as well as increasing adherence to medical advice among adolescents with chronic illness.^26^ Others have advised such technology may be a key aspect of empowering adolescent patients into adulthood as they can be accessed at low or no cost,^27^ an important factor as financial strain was a key transition barrier in our previous study.^18^ Having a patient portal for AYA to access their own health information, communicate with their care providers, and arrange appointments may aid to improve self-management and self-advocacy skills during the transition.^28^

Increasing language support may be one of the most important and complicated aspects of improving the transition success. Although all youth participants reported they themselves had the language skills necessary to understand providers, they explained their parents often faced challenges. Caregiver interviews echoed this. There is a large breadth of work investigating language barriers to service access in Canada and other host countries. This work has called for the translation of online information^29^ and the utilization of informal sources of information dissemination.^30^

Participants advised language supports, such as interpretation, should be provided during appointments and extend beyond. One way this support may be extended beyond appointments is through the provision of family case workers who are able to bridge language barriers and provide additional information and support. Case workers may be especially appropriate for newcomer families who are unsure how to navigate Canadian healthcare systems. Though past research has noted inequities in healthcare access between immigrant and non-immigrant Canadians,^31^ there has yet been a lack of attention on the role of designated and accessible case workers. Future work should examine the outcomes of such an intervention in supporting immigrant and refugee healthcare access broadly as well as transition specifically.

Participants also reported a need for increased service provision and collaboration across service sectors including health, education, settlement, and social work. The World Health Assembly has signaled a commitment to intersectoral policy-making and cross-sector collaboration through the adoption of Health in All Policies (HiAP) approaches.^32^ Some analysis has criticized Canada for falling behind such global health equity initiatives,^33^ particularly for the exclusion of migrants without status from healthcare access.^34^ We offer further evidence to these concerns while highlighting possibilities to connect health and educational supports for adolescents who are navigating transition of health systems and transition to post-secondary schooling simultaneously.

At Mahan et al.^22^’s (2017) third stage, patients can reach their transition goal through a full transfer of care into the adult system. Research shows this third stage remains elusive to many immigrant and non-immigrant patients in Canada.^35^ Thus, this study highlights the significant need for policy and service intervention in support of healthcare transition success.

This study has limitations. Most participants were from the province of Alberta and may not reflect the experience of immigrants and refugees in other parts of Canada. We had participants having many different first languages and countries of origin. Accordingly, it was not possible to make conclusions about similarities/differences based on language and country of origin. The number of service providers was relatively low.

## 5 Conclusion

Our findings fill a gap in knowledge about immigrant and refugee youths’ transition experiences in Canada by centering the voices of patients and their families. This article builds on our previous work from the same data set^18^ which explored barriers and facilitators of successful transition among immigrant and refugee adolescents. The current paper offers recommendations for mitigating barriers to successful transition that immigrant and refugee youth may face. These recommendations can inform future policy formation and service provision to break down barriers to successful transition and better support AYA access to care.

## Funding statement

This research has been funded by the 10.13039/100010322Stollery Children’s Hospital Foundation through the Women and Children’s Health Research Institute under grant #2390.The funding source was not involved in study design; in the collection, analysis and interpretation of data; in the writing of the report; or in the decision to submit the article for publication.

## Ethical statement

The study received approval from the University of Alberta research ethics board on October 21, 2019 and all participants provided informed consent prior to participation. This study was performed in compliance with relevant laws and institutional guidelines.

## CRediT authorship contribution statement

**Andrew S. Mackie:** Writing – review & editing, Supervision, Methodology, Funding acquisition, Conceptualization. **Bukola Salami:** Writing – review & editing, Supervision. **Siciida Ibrahim:** Writing – review & editing, Investigation, Formal analysis. **Michael Kariwo:** Writing – review & editing, Investigation, Formal analysis. **Alyssa Chappell:** Writing – review & editing, Visualization, Supervision, Resources, Project administration, Investigation, Data curation. **Mia Tulli-Shah:** Writing – original draft, Investigation, Formal analysis.

## Declaration of Competing Interest

The authors declare that they have no known competing financial interests or personal relationships that could have appeared to influence the work reported in this paper.

## Data Availability

The data that has been used is confidential.
